# Practical Notes and Formulæ

**Published:** 1882-11-20

**Authors:** 


### PRACTICAL NOTES AND FORMULÆ.

**Torpor of the Colon.**—Dr. H. C. Ghent, (Class of 1856), Belton, Texas.—A few days ago, in looking over one of my old note-books, I found the following: "Jefferson Clinic, January 9th, 1856, service Prof Dunglison, case No. 6. Male adult.

℞ Magnes. sulph ℥ j,

Potass. bitart ʒ j,

Ferri sulph ℈ ss.

M. Sig. For a quart of water. Dose: A wineglassful every morning before breakfast.

Prof. D. then remarked that he often added to the foregoing:

℞ Subcarb. iron grs. xv. M.

This he called the Ferro-saline. The above prescription, in varying proportions, I have used thousands of times during the past quarter of a century, and can safely say that in nineteen cases out of twenty, with the happiest results. I have prescribed it, when indicated, for most all ages, male and female, but there is one class of patients to which, as Dr. D. taught, it is best suited, viz: during adult female life, where there is anæmia with torpor of the colon or habitual constipation. For a case of this character, my usual prescription is the following—

℞ Magnes. sulph ℥ ij,

Potass. bitrat ℥ j,

Ferri sulph ℈ ij,

Tinct. aurantii cort ℥ iv,

Aquæ q. s. ad ℥ xxxij.

M. Sig. Wineglassful every morning before breakfast.

No one prescription from any teacher or professional friend has ever been of so much service to me in the treatment of the above class of cases. I often add the subcarbonate of iron in larger proportion than the original. The dose is not so nauseous as one would imagine from its composition.—*College and Clinic Record.*

**Remedy for Asthmatic Paroxysms**—

℞ Tict. lobeliæ ℥ ij,

Ammon. iodid ʒ iij,

Ammon. bromide ʒ iv,

Syrup tolu ℥ iv.

M. Sig. Teaspoonful every one to four hours.—*Bartholow.*

**Dysmenorrhœa.**—

℞ Tinct. cimicifuga }
Cimicifugæ } aa ʒ i,
Spec. tinct. pulsatilla }
Aqua dist ℥ iv.

M. Sig. Teaspoonful four times a day.—*Med. Brief.*

**Nervine and Anti-Spasmodic—**

| | | |
|---|---|---|
| ℞ | Potassii bromidi | grs. x, |
| | Tinct. conii | gtt. xxx, |
| | Tinct. val. ammoniæ | gtt. xx, |
| | Aquæ camph | ℥ j. |

M. A favorite prescription in the Hospital of Chest diseases, London. It is useful in epilepsy, dysmenorshœa, chorea, hysteria and the like.—*Med. Summary.*

**Apthous Sore Mouth in Infants.**—Prof. Wallace (College and Clinical Record recommends the following:

| | | |
|---|---|---|
| ℞ | Sodii sulphitis | gr. xxx, |
| | Glycerine | aa ℥ ss. |
| | Aqua | |

M. To be used on a swab every two hours. Scrupulous cleanliness is required when a nursing bottle is used. The rubber nipple should be turned inside out after each using, washed clean and kept in a solution of baking soda until again needed. It is better to have two nipples, and to use them alternately. Milk must not be allowed to stand in the bottle till it grows sour.—*American Medical Journal.*

**Treatment of Eczema of the Genitalia, Pruritus and Leucorrhœa.**—In cases of eczema, in which glyceroles and unguents have failed, the following formula has been successful:

| | | |
|---|---|---|
| ℞ | Chlorate of potassium | 30 grammes. |
| | Wine of opium | 50 grammes. |
| | Pure water | 1 quart. |

Applied to the parts by linen compresses covered with oiled silk. If there is much inflammation, precede this with warm hip baths and cataplasms sprinkled with powdered carbonate of lime. In obstinate pruritus, associated with leucorrhœa, a tablespoonful of mixture of equal parts of tincture of iodine and iodide of potassium, in a quart of warm tar-water (tar-water holding the iodine in solution) used daily, night and morning, removes the pruritus and ameliorates the leucorrhœa. In fœtid leucorrhœa two or three tablespoonfuls (in a quart of warm water morning and evening, as an injection) of the following formula will be found useful—

| | | |
|---|---|---|
| ℞ | Chlorate of potassium | 12 grammes, |
| | Wine of opium | 10 " |
| | Tar-water | 300 " |

Or,

| | | |
|---|---|---|
| ℞ | White vinegar (or wine) | 300 grammes, |
| | Tinct. eucalyptus | 45 " |
| | Acid salicylic | 1 " |
| | Salicylate of soda | 20 " |

One to five teaspoonfuls in a quart of warm water as an injection two or three times a day.—*Med. Summary.*

**Boracic Acid Ointment.**—M. J. L. Championniere recommends an ointment made of vaseline and boracic acid as an antiseptic mixture which can be preserved indefinitely and is of great value, being non-irritating.

It forms a bland ointment suitable for superficial ulcers or wounds which are not to be irritated; it is applied on a cloth, on salicylated or absorbent cotton batting.

It can be used with advantage as an application for eczema and intertrigo, which, although not parasitic, gives rise to lesions containing and keeping them.

There is no better topical remedy for the erythema of the buttocks of infants. It is an ointment always clean and aseptic to grease the finger and instruments. Wherever there is an irritated wound, it is a most valuable topical application.

Boracic acid is a less energetic antiseptic than carbolic acid; but its action is nevertheless powerful.

The author has successfully employed it in very fœtid eczemas, and in fœtid sweating of the feet. After washing the feet, the ointment is applied in the interdigital spaces; the effect is very good.

The following is the formula of the ointment—

Boracic acid, finely powdered ................ 1 part,

Vaseline .................................. 2 parts.

The acid must be very finely powdered and sifted and not dissolved in glycerine or alcohol, as this renders the mixture irritating.—*Jovr. de Med. et de Chirurg Prat.—St. Louis Med. and Surg. Journal.*

**A Specific for Singultus.**—This very common affection, of infants and children especially, has a specific remedy, at least one which I have never known to fail. Moisten granulated sugar with good cidar-vinegar; give to an infant from a few grains to a teaspoonful. The effect is almost instantaneous, and the dose seldom needs to be repeated. I have used it for all ages, from infants a few months old to those on the down-hill side of life. Try it.—*Henry Tucker, M. D.*

**Dr. Yuna's Vegetable Liver Pill.**—The following is a good vegetable liver pill—

℞ Leptandrin ......................... ½ scruple,

Podophyllin......................... ½ "

Ext. belladon ........................ ½ "

Ext. nux vom ........................ ½ "

Pulv. ipecac ......................... 5 grains.

M. ft. pil. 30. Sig. One, two or three times daily.—*Druggist's Circular.*

